# Relationship of Self-Rated Health to Stroke Incidence and Mortality in Older Individuals with and without a History of Stroke: A Longitudinal Study of the MRC Cognitive Function and Ageing (CFAS) Population

**DOI:** 10.1371/journal.pone.0150178

**Published:** 2016-02-29

**Authors:** Nahal Mavaddat, Rianne van der Linde, Richard Parker, George Savva, Ann Louise Kinmonth, Carol Brayne, Jonathan Mant

**Affiliations:** 1 Primary Care Unit, Department of Public Health and Primary Care, Strangeways Research Laboratory, Worts Causeway, Cambridge, United Kingdom, CB1 8RN; 2 Department of Public Health and Primary Care, Institute of Public Health, University of Cambridge, University Forvie Site, Robinson Way, Cambridge, United Kingdom, CB2 0SR; 3 Health Services Research Unit, Usher Institute of Population Health Sciences and Informatics, University of Edinburgh, Edinburgh, United Kingdom; 4 School of Health Sciences, University of East Anglia, Norwich Research Park, Norwich, United Kingdom, NR4 7TJ; University of Glasgow, UNITED KINGDOM

## Abstract

**Introduction:**

Poor self-rated health (SRH) has been associated with increased risk of death and poor health outcomes even after adjusting for confounders. However its’ relationship with disease-specific mortality and morbidity has been less studied. SRH may also be particularly predictive of health outcomes in those with pre-existing conditions. We studied whether SRH predicts new stroke in older people who have never had a stroke, or a recurrence in those with a prior history of stroke.

**Methods:**

MRC CFAS I is a multicentre cohort study of a population representative sample of people in their 65th year and older. A comprehensive interview at baseline included questions about presence of stroke, self-rated health and functional disability. Follow-up at 2 years included self-report of stroke and stroke death obtained from death certificates. Multiple logistical regression determined odds of stroke at 2 years adjusting for confounders including disability and health behaviours. Survival analysis was performed until June 2014 with follow-up for up to 13 years.

**Results:**

11,957 participants were included, of whom 11,181 (93.8%) had no history of stroke and 776 (6.2%) one or more previous strokes. Fewer with no history of stroke reported poor SRH than those with stroke (5 versus 21%). In those with no history of stroke, poor self-rated health predicted stroke incidence (OR 1.5 (1.1–1.9)), but not stroke mortality (OR 1.2 (0.8–1.9)) at 2 years nor for up to 13 years (OR 1.2(0.9–1.7)). In those with a history of stroke, self-rated health did not predict stroke incidence (OR 0.9(0.6–1.4)), stroke mortality (OR 1.1(0.5–2.5)), or survival (OR 1.1(0.6–2.1)).

**Conclusions:**

Poor self-rated health predicts risk of stroke at 2 years but not stroke mortality among the older population without a previous history of stroke. SRH may be helpful in predicting who may be at risk of developing a stroke in the near future.

## Introduction

With an ageing population, the burden of stroke, already a significant cause of disability, is expected to rise. [1] It is relevant to identify predictors of stroke incidence and outcome to determine whether they might have any implications for stroke prevention and management.

Self-rated health (SRH) is a subjective assessment of overall health that has been shown to be an independent predictor of all-cause and disease-specific mortality, even after adjusting for objective biological measures and chronic disease. [2–4] It is speculated that the association of poor self-rated health with all-cause mortality may be driven by its association with cardiovascular diseases, in particular stroke.[5] SRH predicts incidence and death from cardiovascular diseases after adjusting for traditional cardiovascular risk factors and pre-existing disease. [6] However, few studies have reported the association of SRH with stroke incidence or mortality and those that do, have not always adjusted for important confounders such as disability, comorbidity and health behaviours. [7–9] It has been suggested that SRH may be particularly predictive of health outcomes in those with pre-existing conditions. [10] For example, Idler et al found SRH predicted all-cause mortality more strongly in those with pre-existing cardiovascular disease. [10] Similarly, Hillen et al found that a measure of comparative SRH at 3 months post-stroke predicted increased risk of recurrence in stroke survivors. [11] In individuals, stroke has been reported to contribute to greater losses in SRH status compared to other chronic conditions. [12] The relationship of SRH with mortality in stroke survivors is therefore of particular interest. Our aim was to determine whether SRH predicts stroke outcomes in older people with and without a prior history of stroke independent of disability levels, other comorbidities and health behaviours. The study used data from the first MRC Cognitive Function and Aging Study (MRC CFAS I), a study of older people aged 65 years and over recruited from the community. MRC CFAS I participants underwent physical, psychological, social and cognitive assessments at baseline, with follow-up including self-reported stroke after two years. Notifications of cause-specific mortality were received for up to 13 years.

## Methods

The MRC CFAS I is a multi-centre population-representative study of individuals aged 65 years and over (including those living in care homes). The study began in 1991 and was designed to estimate the prevalence and incidence of dementia as described elsewhere. [13]

The study has six centres across England and Wales chosen to represent the national variation of urban-rural mix, socio-economic deprivation and rates of chronic disease. [13] Five of these centres with identical study designs (Oxford, Nottingham, Cambridgeshire and Gwynedd) are used in the present investigation. The sixth centre (Liverpool) used a different design and is not included in the present study. Random samples of people in their 65th year and above were obtained from Family Health Service Authority lists (the agency responsible for maintaining registers of general practice populations at that time) from these five centres. The sample was stratified by age (65–74 years and 75 years and over) and equal numbers were randomly selected from these two age groups with the aim of recruiting 2500 to each centre.

Of those 16258 eligible and available to take part in CFAS, 13004 (80%) agreed to participate. All study centres obtained ethical approval from local research committees (Oxford Research Management & Governance, Nottingham Research Ethics Committee 1, Cambridge City Research Ethics Committee, East Cambs & Fenland Local Research Ethics Committee, North West Wales NHS Trust Research Governance Committee) and from the Eastern Multicentre Research Ethics Committee in the United Kingdom Ref: 05/MRE05/37. Eligible participants (or their proxies where appropriate) provided informed written consent.

### Baseline Information

Trained interviewers undertook baseline interviews in the participants’ homes including assessments of socio-demographic characteristics, and disease history including previous stroke, coronary heart disease and diabetes (full details at www.cfas.ac.uk).Socio-demographic factors collected included age, sex, marital status, type of accommodation and social class using the Registrar General’s Occupational Classification with the detailed classification described elsewhere. [14]

The presence of stroke was determined from self-report through the question: “Have you ever had a stroke that required medical attention?”

General subjective health status or self-rated health (SRH) was determined with the question: “Would you say that for someone of your age, your own health in general is” followed by a list of four options: poor, fair, good or excellent.

Participants were asked about health behaviours including smoking status and alcohol intake. Comorbidities were assessed by asking: “Have you ever suffered from high blood pressure/angina/heart attack/diabetes/head-injury?” with each assessed in a separate question.

Functional status was determined by enquiring about activities of daily living (ADL) with ADL disability defined as requiring help at least several times per week with activities of daily living such as washing, cooking for him or herself, dressing, or if the respondent was housebound. Impairment of Instrumental activities of daily living (IADL) was determined as needing help with heavy housework or shopping and carrying heavy bags. Cognitive status was measured using the Mini Mental State Exam (MMSE). [15]

### Follow-up

At 2 years, re-screening interviews took place. At this time, the presence of non-fatal stroke was determined from self-report through the question: “Have you had a stroke in the last two years?”

All participants were flagged with the NHS Central Register. Information about date of death and stroke deaths was available in the CFAS until 31st December 2004.

Strokes recorded as either primary or underlying cause of death both (i) between baseline interview and the planned 2 year follow-up and (ii) at any time before December 2004, were defined using the ICD-9 codes 430–438 on death certification for each participant.

### Analysis

To determine whether SRH predicts stroke outcomes in older people, we asked two questions about those with and without a history of stroke:

1. What is the association between baseline SRH, and stroke incidence and mortality at 2 years after baseline interview?
2. What is the association between baseline SRH and stroke mortality and survival at 13 years follow-up?

All analyses were performed using Stata 11.1. Any participants who had missing data regarding presence of stroke or SRH or had an MMSE that was missing or less than 17 (as their responses could not be considered reliable for the purposes of this analysis), were excluded from the analyses at baseline.

The distribution at baseline of demographic, physical and social characteristics were described for participants with and without stroke. These included age group (65–74, 75–84, and 85+), sex, social class (divided into manual (IIIb, IV, V) and non-manual (I, II, IIIa)), and ADL (No impairment, impairment of IADL, impairment of ADL).

Two year incident stroke was defined by self or informant report at follow-up, or death from stroke (as primary or underlying cause) on the death certificate before the planned two year follow-up could take place.

Inverse probability weights were used throughout to ensure the sample was representative of the target population. Weights were estimated with logistic regression using presence in each phase of the study as an outcome and taking into account over-sampling of over-75s at baseline. Estimation of attrition weights for incident stroke included those who died during follow-up. Two year estimates of stroke incidence are therefore applicable to the whole of the population. The odds of having a stroke event or death from stroke were calculated at 2 years using logistic regression models. For these, SRH was dichotomised into two groups (‘excellent/ good’ or ‘fair/poor’).

Survival time until 2004 was calculated by subtracting the date of the screening baseline interview and date of death. If still alive, the time between the interview and the end of the follow-up period was used. The risk of death from all-causes and from stroke for both individuals with and without history of stroke were calculated using Cox Proportional Hazard models after testing the proportional hazard assumption (which was not violated) using the log-cumulative hazard plot and by comparing the predicted survival plot to the Kaplan-Meier plot. Death from other causes was treated as a censoring event.

Regression models and survival models were adjusted for baseline socio-demographic factors: sex, age group, marital status, social class; health-behaviour: smoking, alcohol drinking and co-morbidities: high blood pressure, angina, heart attack, diabetes, head injury and disability

## Results

Of those 16258 eligible and available to take part in CFAS, 13004 (80%) agreed to participate. 138 (1.1%) had information about stroke missing and were excluded from this analysis. 889 (6.9%) who had an MMSE missing or equal or below 17, (706 (5.9%) without stroke and 183 (19.0%) with stroke) were also excluded from the analysis. In addition, 20 participants (16 with stroke, 4 without stroke) who had missing data on SRH were excluded from analyses. In total 1,047 (8.0%) of participants were excluded from this analysis, leaving 11,957 eligible of which 11,181 (93.8%) had no stroke and 776 (6.2%) had one or more previous strokes.

At 2 years, 8,827 persons participated in the follow-up interview. By the end of 2004, 4878 participants (40.8%) included in the analysis were alive.

The baseline distribution of demographic, physical and social characteristics for participants with and without stroke have been previously fully reported. [16] Participants with stroke were older, more likely to be male, disabled, and have lower SRH (Table 1). As reported previously, people with stroke were more likely to be of lower social class, former smokers, and to have comorbidities. [16]

**Table 1:** Distribution of demographic variables in those with and without stroke in the population of England and Wales aged 65 years and older.

	Individuals with stroke		Individuals without stroke	
	N	%*	N	%*
**All**	776	6.2	11,181	93.8
Sex				
Females	400	50.6	6,674	59.0
Males	376	49.4	4,507	41.0
**Age**				
Mean years	76.2	6.6	74.7	6.6
**Self**-**rated Health**				
Excellent	59	7.5	2,349	21.3
Good	280	36.0	5,535	49.7
Fair	319	40.9	2,774	24.4
Poor	118	15.6	523	4.7
**Disabilities**				
None	333	44.9	8305	76.4
IADL †	130	16.4	1557	13.2
ADL †	311	38.8.	1292	10.4

* Percentages backweighted to normal population

† IADL—impairments of instrumental activities of daily living ADL–impairments of activities of daily living

2.3% of those without a history of stroke included in the analysis, experienced a stroke event by two years compared to 14.3% of those who had a history of stroke, while 0.9% died from their stroke within this time in those without a history of stroke compared to 4.8% with a history of stroke. (Table 2) By the end of 2004, 8.2% of those without a prior history of stroke and, 25.8% with a prior history of stroke experienced a fatal stroke.

**Table 2:** Non-fatal and fatal stroke, survival and mortality at 2 years and follow-up until 30 December 2004 in those with or without stroke at baseline by self-rated health.

Baseline	2 year Follow-up					Until 30 December 2004			
	No event	New stroke (first or recurrence not including fatal events)	Died with stroke as primary or underlying cause	Died because of other causes	Missing†	Alive at end of the follow-up period	Died without stroke	Fatal stroke	Mean follow-up
	N (%)*	N (%)*	N (%)*	N (%)*	N (%)*	N (%)*	N (%)*	N (%)*	Years
**Total**	**8,191 (67.6)**	**245 (2.0)**	**140 (1.1)**	**853 (6.8)**	**2,528 (22.6)**	**4878(40.8)**	**5887 (49.2)**	**1192 (10.0)**	
***No prior history of stroke***									
All	*7*,*757 (68*.*4)*	*168 (1*.*4)*	*103 (0*.*9)*	*767 (6*.*5)*	*2*,*386 (22*.*8)*	*4*,*746 (44*.*9)*	*5*,*447 (46*.*9)*	*988 (8*.*2)*	9.3
**Self-rated health**									
Excellent (n = 2349)	1,702 (71.7)	20 (0.7)	21 (0.8)	97 (3.8)	509 (23.0)	1,264 (56.7)	905 (36.5)	180 (6.9)	10.0
Good (n = 5535)	3,997 (71.4)	85 (1.5)	43 (0.7)	306 (5.2)	1,104 (21.3)	2,524 (48.2)	2,508 (43.5)	503 (8.3)	9.5
Moderate (n = 2774)	1,771 (62.8)	57 (2.0)	32 (1.1)	276 (9.5)	638 (24.7)	847 (33.0)	1,663 (58.0)	264 (9.0)	8.4
Poor (n = 523)	287 (53.8)	6 (1.2)	7 (1.2))	88 (15.9)	135 (27.9)	111(22.7)	371(69.2)	41(8.1)	7.5
***Prior history of stroke***									
All	*434 (55*.*5)*	*77 (9*.*5)*	*37 (4*.*8)*	*86 (11*.*0)*	*142 (19*.*3)*	*132 (18*.*1)*	*440 (56*.*2)*	*204 (25*.*8)*	7.0
**Self-rated health**									
Excellent (n = 59)	38 (63.1)	5 (8.4)	4 (7.0)	3 (4.7)	9 (16.8)	11 (17.9)	30 (55.1)	18 (26.9)	7.1
Good (n = 280)	115 (55.0)	34 (11.6)	9 (3.7)	26 (8.9)	56 (20.8)	60 (22.7)	147 (52.0)	73 (25.4)	7.4
Moderate (n = 219)	183 (56.7)	26 (8.0)	17 (4.9)	40 (12.8)	53 (17.6)	51 (17.4)	189 (57.8)	79 (24.8)	6.9
Poor (n = 118)	58 (50.0)	12 (9.2)	7 (6.2)	17 (13.4)	24 (21.3)	10 (9.8)	74 (61.4)	34 (28.7)	6.1

*Percentages backweighted to normal population

†Not interviewed at two years, but death data available

Table 3 shows that among those with no prior history of stroke, odds of stroke and all-cause mortality at 2 years were significantly higher in those with ‘fair/poor’ compared to ‘excellent/good’ SRH, after adjusting for potential confounding factors including socio-demographic factors, health-behaviours, co-morbidities and disability. The odds of death from stroke at 2 years were not significantly associated with SRH in people with no stroke history, although only 103 individuals in this group suffered a fatal stroke within two years.

**Table 3:** Odds of stroke and all-cause mortality with poor SRH at 2 years in those with or without a prior history of stroke* ^†^

	Odds of non-fatal stroke		Odds of fatal and non-fatal stroke		Odds of fatal stroke		Odds of all-cause mortality	
	Model	Adjusted Model	Model	Adjusted Model	Model	AdjustedModel	Model	Adjusted Model
	OR (95% CI)	OR (95% CI)	OR (95% CI)	OR (95% CI)	OR (95% CI)	OR (95% CI)	OR (95% CI)	OR (95% CI)
**No prior history of stroke**	**1.7 (1.2–2.3)**	**1.5 (1.2–1.9)**	**1.4 (1.0–1.8)**	**1.5 (1.1–1.9)**	**1.5 (1.0–2.3)**	1.2 (0.8–1.9)	**2.4 (2.0–2.7)**	**1.7(1.4–2.0)**
**Prior history of stroke**	0.8 (0.5–1.3)	0.8 (0.4–1.3)	0.9 (0.6–1.4)	0.9 (0.6–1.4)	1.4 (0.7–2.9)	1.1 (0.5–2.5)	**1.6 (1.1–2.4)**	1.1 (0.7–1.8)

*Adjusted for socio-demographic factors: sex, age group, marital status, social class; health-behaviour: smoking, alcohol drinking and co-morbidities: high blood pressure, angina, heart attack, diabetes, head injury, disability

† Reference: ‘excellent/good’ versus ‘fair/poor’

There was no significant association of SRH at baseline with stroke incidence, stroke death or of all-cause mortality at 2 years among those with a previous history of stroke.

In univariate analysis among those with no stroke history, there was an increased risk of stroke mortality among those with fair or poor SRH at 13 year follow up (Table 4). This was largely attenuated in multivariate models with little evidence for an independent effect of SRH on stroke specific mortality. In those with a prior history of stroke there was no association of SRH with stroke specific mortality in the longer term.

**Table 4:** Univariate and adjusted hazard ratios of 13 year survival from fatal stroke by self-rated health.*

Self-rated health	Total participants at baseline	Alive or died because of other causes	Died with stroke as primary or underlying cause	HR (95% CI)Univariate	HR (95% CI) Adjusted for confounders
No prior history of stroke					
Excellent	2349	2169	180	1	1
Good	5535	5032	503	1.3(1.1–1.5)	1.2(1.0–1.4)
Fair	2774	2510	264	1.7(1.4–2.0)	1.3(1.1–1.6)
Poor	523	482	41	1.7(1.2–2.4)	1.2(0.9–1.7)
Prior history of stroke					
Excellent	59	41	18	1	1
Good	280	207	73	0.8 (0.5–1.4)	0.8 (0.5–1.3)
Fair	319	240	79	0.9(0.5–1.5)	0.8 (0.4–1.3)
Poor	118	84	34	1.2(0.7–2.2)	1.1 (0.6–2.1)

*Adjusted for socio-demographic factors: sex, age group, marital status, social class; health-behaviour: smoking, alcohol drinking and co-morbidities: high blood pressure, angina, heart attack, diabetes, head injury, disability

## Discussion

This study confirms that in the older population without a history of stroke, premorbid SRH independently predicts stroke incidence and all-cause mortality at 2 years. However, there was no evidence of a significant association between SRH and death from stroke at 2 years or fatal stroke after a 13 year follow-up period in this group. In the older population who had previously suffered a stroke, no significant relationship was found between SRH and stroke recurrence or death, or stroke survival.

### Comparison with previous studies

#### SRH and stroke incidence–non-stroke population

Two previous studies have looked at the association of SRH with stroke incidence in unselected patient populations. Emmelin et al (2003) in an incident case-control study nested within the Vasternotten Intervention Programme and the Northern Sweden MONICA matched 473 cases with stroke with controls without known stroke history and found that after adjusting for diabetes and cardiovascular risk factors, SRH independently predicted the risk of stroke (death and non-fatal stroke combined) specifically for men with poor SRH who had a fourfold stroke risk compared with controls. [2] An analysis of 4770 mid-life adults participating in the US Health and Retirement study also found, after controlling for risk factors and health care utilisation, a significant association between SRH and first onset of stroke based on self-report (HR 1.54 p< = .001), [17] of a strength similar to our own in older adults.

#### SRH and stroke related mortality–non-stroke population

**Fig 1:**
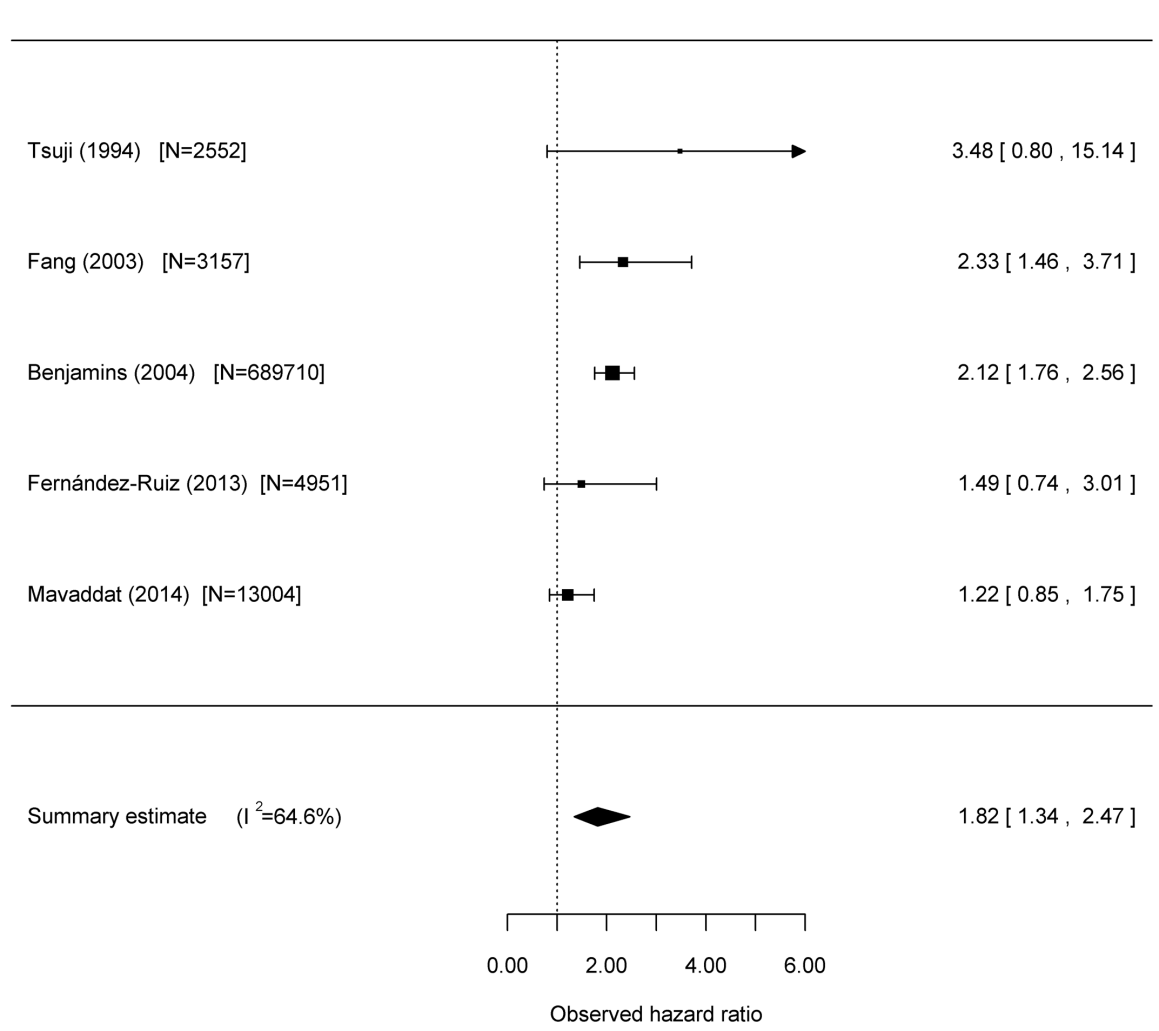
Meta-analysis of fatal stroke events in unselected populations with varying degrees of control for CVD status and risk factors: ‘poor’ health relative to ‘good/excellent’ health

In the course of a systematic review and meta-analysis of studies reporting the relationship between SRH and cardiovascular disease outcomes (full description reported elsewhere), [6] we also identified three longitudinal studies that reported on fatal stroke outcome in unselected patient populations, which we used as a basis for a separate meta-analysis for this paper as well as a further study published after the systematic review. Details of the studies are found in Table 5, and Fig 1 summarises the results of the meta-analysis including our own study, for the non-stroke population. Tsuji et al (1994) and Fernández-Ruiz (2013) also studied older people. Tsuji et al had a follow-up period of 3 years, [7] Fang et al (2003) 8 years, [18] and Benjamins et al (2004) by far the largest study, 7 years, [19] while Fernández-Ruiz had a long follow-up similar to our study of 13 years. [5] While we analysed those with a prior history of stroke separately, Tsuji excluded those with baseline coronary heart disease and stroke from their study.

**Table 5:** Characteristics of studies included in meta-analysis.

Study & **year published**	Country	Study Sample	Years follow up	SRH question (number of response options)	Population Baseline CVD status	Baseline CVD risk factors & measurement	Analysis adjustments
Mavaddat (2014)	UK	N = 11,181 41% males Mean age 74.7	13 (total)	(4) excellent vs poor	Reported and those with stroke excluded	Demographics Risk Factors	Age, sex, marital status, social class; health behaviours, ADL, co-morbidities
Fernández-Ruiz (2013)	Spain	N = 4598 42.4% males Mean age 77.9	13 (total)	(5) v. good vs v.poor	Not reported	Demographics	Age, sex, depression, MMSE, lifestyle, functional status
Benjamins (2004)	USA	N = 689,710 46.2% males Mean age 44.4	7 (mean)	(5) v. good vs poor	Not reported	Demographics, BMI	Age, sex, race, education, marital status, family income, employment status
Fang (2003)	China	N = 3,157 48.7% males	8 (total)	(3) excellent vs poor	Reported Baseline CVD status but not risk factors	Demographics, BMI, BP	Age, sex, marital status, residence, hospitalisation history during previous year, history of disease ADL, BMI, mental state and depression
Tsuji (1994)	Japan	N = 2,552 44.6% males Aged 65–113 at recruitment	2.9 (mean)	(4) excellent and good vs poor	Reported Baseline CHD and stroke excluded	Demographics, diabetes, hypertension	Age, sex, ADL disability, ambulatory activity, use of medical services

**Key:** SRH = self-rated health; BMI = body mass index; AMI = acute myocardial infarction; BP = blood pressure; SBP = systolic blood pressure; HDL = high density lipoprotein; LDL = low density lipoprotein; ADL = activities of daily living; MMSE = mini-mental state examination

Our meta-analysis suggests an overall significant relationship between SRH and stroke mortality (HR1.8 (1.3–2.5)). However, there was significant heterogeneity and a qualitative difference in results between studies.

Based on our findings and that of Fernández-Ruiz, it may be that any relationship of SRH with stroke mortality is not as strong in older as compared to younger people. However, our study also had better control of confounders including better measurement of self-report health behaviours, comorbidities and levels of physical disability than the studies involving younger participants. Compared to that of Fernández-Ruiz our study also controlled for comorbidities that serve as risk factors for stroke and we excluded all participants with a previous stroke from our analysis. Tsuji et al and Fernández-Ruiz also controlled for functional ability in their study and found no significant relationship between SRH and stroke mortality. There is a strong relationship between SRH and levels of physical disability, [3, 20] and it may be that in older people, any relationship between SRH and stroke mortality found in other studies may have been mediated by disability level, itself a risk factor for stroke. [21]

#### SRH and stroke-related mortality–people with previous stroke

Hillen et al (2003) is the only study to investigate the association of SRH with stroke recurrence in people with previous stroke. [11] They found, like us, that current SRH status did not relate to recurrence-free survival, but also that SRH transition was associated with stroke recurrence in those with change to worse SRH status. It has been hypothesised that SRH may be more predictive of mortality in those with pre-existing conditions, partly because the presence of a diagnosis may influence self-ratings. Idler et al. found that SRH predicts subsequent mortality more strongly in those with circulatory system disease than in those with no identified cardiovascular condition. [10] In our previous meta-analysis (which included Idler et al), we did find a significant association of SRH with CVD mortality in people with existing CVD, but these studies had not adjusted for disability levels and were in younger populations. [6] A possible explanation for our different finding in stroke survivors is that although those with more severe cognitive deficits were excluded in our study, it is possible that some were less able to adequately respond to the SRH question or used different measures by which to assess their health compared to those without physical or cognitive disabilities. Alternatively, it is plausible that the presence of stroke overwhelms the predictive effects of SRH on further stroke events and death.

### Study limitations

Non-fatal stroke data in CFAS were dependent on self or proxy report, and some older people may have omitted reporting a milder stroke or reported a stroke in the absence of a confirmed diagnosis, thus potentially under or overestimating any relationship between SRH and stroke outcome. Baseline and two-year follow-up were carried out in the 1990s and changes in rates of vascular events or survival from vascular events may have occurred since then. Detailed cardiovascular risk factors were not available in this study and not all comorbidities were adjusted for. For example the presence or absence of atrial fibrillation was not recorded.

## Conclusion

This study confirms that in the older population without a history of stroke, SRH predicts overall mortality. Consistent with this there is a small but significant independent relationship between poor SRH and stroke incidence. However there is no relationship between SRH and stroke mortality in the short or longer term in the older population. In older people with a history of stroke, there is no relationship between SRH and stroke outcomes.

While in older stroke survivors, SRH does not predict future stroke events, it may nevertheless have other prognostic value. The relationship of SRH with health outcomes such as institutionalisation and functioning in stroke survivors warrants further study. For the older population without a history of previous stroke, SRH may be helpful in predicting who may be at risk of developing a stroke in the near future. Self-rated health is a multidimensional construct, an “effective summary” of multiple measures and dimensions of health incorporating not only the presence of disease and symptoms but a wide range of lifestyle and psychosocial predictors. [20, 22–24] We have previously reported on determinants of self-rated health in older individuals with or without a stroke in the MRC CFAS 1 where a number of modifiable factors such as ‘presence of depression’ and ‘getting out and about’ were identified. [16] Increased attention to screening and addressing these and other reasons for poor SRH in the older population may therefore be warranted.
